# A potential founder variant in *CARMIL2/RLTPR* in three Norwegian families with warts, molluscum contagiosum, and T‐cell dysfunction

**DOI:** 10.1002/mgg3.237

**Published:** 2016-09-17

**Authors:** Hanne S. Sorte, Liv T. Osnes, Børre Fevang, Pål Aukrust, Hans C. Erichsen, Paul H. Backe, Tore G. Abrahamsen, Ole B. Kittang, Torstein Øverland, Shalini N. Jhangiani, Donna M. Muzny, Magnus D. Vigeland, Pubudu Samarakoon, Tomasz Gambin, Zeynep H. C. Akdemir, Richard A. Gibbs, Olaug K. Rødningen, Robert Lyle, James R. Lupski, Asbjørg Stray‐Pedersen

**Affiliations:** ^1^ Department of Medical GeneticsOslo University Hospital and University of OsloOsloNorway; ^2^ Department of ImmunologyOslo University HospitalOsloNorway; ^3^ Institute of Clinical MedicineUniversity of OsloOsloNorway; ^4^ Section of Clinical Immunology and Infectious DiseasesOslo University HospitalOsloNorway; ^5^ Research Institute of Internal MedicineOslo University HospitalOsloNorway; ^6^ Department of PediatricsOslo University HospitalOsloNorway; ^7^ Department of Medical BiochemistryInstitute of Clinical MedicineUniversity of OsloOsloNorway; ^8^ Department of MicrobiologyOslo University HospitalOsloNorway; ^9^ Department of PediatricsSørlandet HospitalKristiansandNorway; ^10^ Institute of computer scienceWarsaw University of TechnologyWarsawPoland; ^11^ Baylor‐Hopkins Center for Mendelian Genomics (BHCMG) of the Department of Molecular and Human GeneticsBaylor College of MedicineHoustonTexas; ^12^ Human Genome Sequencing Center of Baylor College of MedicineHoustonTexas; ^13^ Department of Molecular and Human GeneticsBaylor College of MedicineHoustonTexas; ^14^ Department of PediatricsBaylor College of Medicine, and Texas Children's HospitalHoustonTexas; ^15^ Norwegian National Unit for Newborn ScreeningDivision of Children and Adolescent MedicineOslo University HospitalOsloNorway

**Keywords:** Absence of heterozygosity, *CARMIL2*, exome sequencing, founder variant, lymphocyte function, lymphocyte subpopulation, molluscum contagiosum, primary immunodeficiency, *RLTPR*, warts

## Abstract

**Background:**

Four patients from three Norwegian families presented with a common skin phenotype of warts, molluscum contagiosum, and dermatitis since early childhood, and various other immunological features. Warts are a common manifestation of *human papilloma virus* (HPV), but when they are overwhelming, disseminated and/or persistent, and presenting together with other immunological features, a primary immunodeficiency disease (PIDD) may be suspected.

**Methods and results:**

The four patients were exome sequenced as part of a larger study for detecting genetic causes of primary immunodeficiencies. No disease‐causing variants were identified in known primary immunodeficiency genes or in other disease‐related OMIM genes. However, the same homozygous missense variant in *CARMIL2* (also known as *RLTPR*) was identified in all four patients. In each family, the variant was located within a narrow region of homozygosity, representing a potential region of autozygosity. *CARMIL2* is a protein of undetermined function. A role in T‐cell activation has been suggested and the mouse protein homolog (Rltpr) is essential for costimulation of T‐cell activation via CD28, and for the development of regulatory T cells. Immunophenotyping demonstrated reduced regulatory, CD4+ memory, and CD4+ follicular T cells in all four patients. In addition, they all seem to have a deficiency in IFN *γ* ‐synthesis in CD4+ T cells and NK cells.

**Conclusions:**

We report a novel primary immunodeficiency, and a differential molecular diagnosis to *CXCR4‐*,*DOCK8‐*,*GATA2‐*,*MAGT1‐*,*MCM4‐*,*STK4‐*,*RHOH‐*,*TMC6‐,* and *TMC8‐*related diseases. The specific variant may represent a Norwegian founder variant segregating on a population‐specific haplotype.

## Introduction

Primary immunodeficiencies are clinically characterized by an increased rate of infections, malignancy, and autoimmunity reflecting immune dysregulation. Patients not only have increased susceptibility to severe infections and infections with uncommon pathogens but also harbor an increased burden of common and clinically less severe infections.

Warts are a common manifestation of *human papilloma virus* (HPV) and will affect most people at some point in their life. However, when they are overwhelming, disseminated, and/or persistent, a primary immunodeficiency disease (PIDD) may be suspected. The immunological mechanisms for controlling HPV infections leading to warts are not yet fully delineated, but adequate T and NK cell immunity are necessary to control and eliminate HPV infection (Leiding and Holland 2012). Mutations in several different immune‐related genes are known to cause the HPV lesion phenotype such as *CXCR4* (MIM:162643), *DOCK8* (MIM:611432), *GATA2* (MIM:137295), *MCM4* (MIM:602638), *STK4* (MIM:604965), *RHOH* (MIM:602037), *TMC6* (MIM:605828), *TMC8* (MIM:605829) (Leiding and Holland 2012; Al‐Herz et al. 2014), and *MAGT1* (MIM:300715) (Stray‐Pedersen et al. 2016), but in many patients, no known predisposing mutation is found. Persistent warts may transform to cancer (Rous and Beard 1935), contributing to the risk of malignant disease in immunodeficient patients. Still, patients with immunodeficiency and warts often have other phenotypic characteristics as well, such as recurrent airway and skin infections, inflammation in the gastrointestinal tract, and eczema. This illustrates that although susceptibility to warts are a common characteristics associated with pathogenic variants in these genes, the patients may also display other distinct phenotypes.

Here, we report a novel primary immunodeficiency caused by biallelic mutations in *CARMIL2* (MIM: 610859) in four patients from three different families. The phenotype is a differential molecular diagnosis to other HPV‐related immunodeficiencies.

## Methods

### Clinical samples

**Figure 1:**
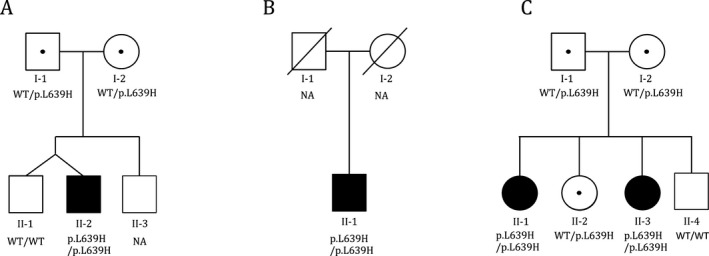
Segregation of the *CARMIL2* p.Leu639His variant in the family of (A) patient #1, (B) patient #2 (parental samples not available), and (C) two sisters in the same family (patients #3 and 4).

Exome sequencing was carried out on the four affected patients (Fig. 1) as part of a larger study to identify genetic causes of primary immunodeficiency (Stray‐Pedersen et al. 2016). Patients #3 and 4 are sisters (Fig. 1C), but the three families are otherwise not known to be related, although they all originate from the southern part of Norway. An overview and comparison of the phenotype of the four affected subjects is shown in Table 1.

**Table 1:** Clinical features of the four patients with homozygous *CARMIL2* mutation

Phenotypes	Patient 1	Patient 2	Patient 3	Patient 4
Warts	+	+	+	+
Molluscum contagiosum	+	+	−	−
Viral skin infections	−	−	VZV	VZV/HSV
Atopic eczema	+	+	−	+
Photodermatitis	−	−	+	++
Hyperkeratosis/psoriasis‐like lesions	+	+	+	+
Dermatophytic lesions	+	+	−	+
Aphthous stomatitis	(+)	−	+	+
Asthma	+	+	(+)	+
Nasal congestion	+	++	−	(+)
Respiratory infections	(+)	−	(+)	+
Lung	COPD	COPD	−	Lung scarring
Short stature (centile)	<< 2.5	>10	>25	>50
Dysuria	−	+	−	+
BK virus cystitis	−	++	−	++
Abdominal findings	Chronic diarrhea and abdominal pain	−	IBD	IBD
Muscle cramps	+	+	−	(+)
Fatigue	−	(+)	(+)	−
Various others	Gastric ulcers in early childhood	−	Leiomyosarcoma	Congenital heart defect, night sweat

VZV, Varicella zoster virus; HSV, Herpes simplex virus; COPD, Chronic obstructive pulmonary disease; IBD, Inflammatory bowel disease; +, feature present (++ to higher or (+) lower degree), −, feature not present.

Patient #1 (family A, individual II‐2) is an 18‐year‐old male with an initial clinical diagnosis of combined immunodeficiency. He is a twin, born at 32 weeks gestational age and was reported to have had an episode with necrotizing enterocolitis at 2 weeks of age. He had asthma‐like symptoms in the first year of life which continued with a severe course through childhood treated with inhaled corticosteroids, and with a clinical course complicated by recurrent upper and lower respiratory infections. His childhood asthma and respiratory infectious problems have greatly improved with age. However, he now has chronic obstructive pulmonary disease (COPD) with radiograpically verified bronchiectasis, and he still has chronic nasal congestion.

He had endoscopically verified gastric ulcers from age two, and later also a duodenal ulcer. Although there was no detection of *H. pylori*, he responded to a course of proton pump inhibitor and antibiotics. He had episodes of aphthous stomatitis while treated with inhaled corticosteroids. Chronic diarrhea and episodes with abdominal pain were reported to be present since childhood. Colonoscopy at age 13 years indicated mild inflammatory changes in the mucosa, but otherwise normal findings. He has had warts since early childhood, especially widespread on the hands, fingers, and on the soles of the feet, as well as molluscum contagiosum on his trunk, legs, neck, and around his mouth. The molluscum contagiosum have also been present on his head together with a generalized desquamation affecting his entire scalp, reminiscent of seborrheic dermatitis/tinea capitis/fungal skin infection. His facial eczema has improved with age, and he is presently not using any corticosteroids. He has short stature likely caused by recurrent infections and chronic abdominal complaints with diarrhea and mild bowel inflammation throughout childhood and adolescence. At 18 years of age, his final height is 163 cm, below the 2.5 percentile and 30 cm lower than his dizygotic twin brother. None of his relatives has a similar medical history and his parents are not known to be related. His two brothers have no reported skin lesions and no abdominal complaints. Genetic testing by Sanger sequencing for *DOCK8, TMC6,* and *TMC8* mutations were negative.

Patient #2 (family B, individual II‐1) is a 52‐year‐old male initially suspected to have hemophagocytic lymphohistiocytosis (HLH), but did not completely fulfill the criteria for HLH (Jordan and Filipovich 2008). Since childhood, he has been affected with atopic eczema and dermatitis with sun exposure, which worsened in his twenties with patches of eczema‐like rash, especially on the trunk. He has hyperkeratosis, psoriasis‐like lesions, and widespread desquamations on his scalp. He has had recurrent fungal skin infections (trichophyton species/tinea corporis/fungal dermatitis), and warts on his hands and fingers, as well as widespread molluscum contagiosum. His main skin problem presently is palmoplantar hyperkeratosis with painful skin fissures. He has been a smoker for many years, has been treated for asthma, and has clinical signs of mild COPD, with no increased frequency of respiratory tract infections. He has nasal congestion with verified polyps. Low‐grade chronic cystitis with urinary tract stenosis and presence of BK virus in the urine has been observed.

Patients #3 and #4 (family C) are two sisters with an initial diagnosis of unspecified T‐cell immunodeficiency prior to exome sequencing. They have two unaffected siblings, and their parents are not known to be related.

Patient #3 (family C, individual II‐3) is the younger sister and now 30 years old. She presented with abdominal pain and diarrhea at 2 years of age, evolving into dysentery and an unspecified colitis leading to subtotal colectomy and ileostomy at age 13. The ileostomy was later converted to an ileoanal anastomosis and, although she has had recurrent problems with stenosis, recently there has been no signs of active inflammation. She has also had unspecified ulcerations of the esophagus, as well as aphthous stomatitis. The patient has a variable degree of psoriatic lesions and seborrheic dermatitis, mainly affecting the scalp, and recurrent bacterial skin infections, occurring in the dermatitis lesions. From age 10, she developed multiple hyperkeratotic warts, mainly affecting the palm of the hands, fingers, toes, and soles, as well as recurrent condyloma. She suffers from frequent bouts of herpes labialis and has had three episodes of shingles. At age 24, she had a malignant leiomyosarcoma behind the right scapula, which was treated with surgery and radiation. In the last year, she has developed signs of UV‐sensitive dermatitis, while not as severe as her sister.

Patient #4 (family C, individual II‐1) is the older of the two sisters and now 37 years old. She experienced increasing numbers of warts affecting her hands, fingers, and feet from age four, and later had recurrent condyloma. Like her younger sister, she suffers from herpes simplex virus (HSV) infections and has had several episodes of shingles. She has been affected with atopic and psoriatic‐like dermatitis since childhood. From her late twenties, she has been increasingly affected with UVA‐sensitive dermatitis with solar urticaria appearing after only a few seconds of sun exposure. At age 25, she was diagnosed with Crohn's disease affecting the esophagus and colon, with recurrent mouth ulcers, fissures, gastrointestinal bleeding, and diverticulosis. She has had asthma with an increased frequency of both upper and lower respiratory tract infections since childhood, and over time has developed hypogammaglobulinemia.

After her first childbirth, she developed an aggressive hemorrhagic BK virus cystitis treated with intravenous immunoglobulins and ciprofloxacin with some effect. She was born with an atrial septal defect surgically closed at age six, and has mitral insufficiency that is kept under observation.

### Exome sequencing

Exome sequencing was performed using genomic DNA extracted from whole blood. Exome sequencing of patients #1 and 2 was performed at the Norwegian Sequencing Centre (www.sequencing.uio.no) in the Department of Medical Genetics, Oslo University Hospital, Norway. For these patients, exome capture was performed using Agilent SureSelect Human All Exon (v.5; Agilent technologies, CA) and sample preparation was performed according to manufacturer's recommendations. The captured exome was sequenced on an Illumina HiSeq 2500 (Illumina, CA) with 100 bp paired‐end reads, resulting in an average coverage of 20× for >90% of the bases in both patients. Reads that did not pass Illumina's standard filter were removed prior to alignment. The remaining reads were aligned to the reference human genome (hg19), using Novoalign (v3.02; Novocraft Technologies, Selangor, Malaysia). The initial alignment files were realigned using the Genome Analysis Toolkit (GATK, v3.1) (Mckenna et al. 2010; Depristo et al. 2011) and PCR duplicates were marked using Picard (v1.88; http://picard.sourceforge.net). Next, base quality scores were recalibrated and variant calling was performed using GATK. ANNOVAR (v2013August23)(Wang et al. 2010) was then used for variant annotation. Finally, HGMD annotations and additional information on PIDD candidate genes were added to ANNOVAR‐annotated files using an in‐house developed annotation pipeline.

Exome sequencing of patients #3 and 4 was performed in Houston at the Human Genome Sequencing Center at Baylor College of Medicine (BCM‐HGSC), as part of the Baylor Hopkins Center for Mendelian Genomics initiative. The exome sequencing pipeline used at BCM‐HGSC has been previously described (Yang et al. 2013). Illumina paired‐end libraries were prepared and exome capture was performed using the in‐house developed BCM‐HGSC Core design and sequenced on the Illumina HiSeq 2500 platform (Illumina). Sequencing data were processed through the HGSC‐developed Mercury pipeline to produce variant call format files (. vcf) using the Atlas2 variant calling method (Li and Durbin 2009); (Li et al. 2009); (Challis et al. 2012); (Danecek et al. 2011). Variants were annotated using the in‐house developed Cassandra annotation pipeline (Bainbridge et al. 2011) based on ANNOVAR (Wang et al. 2010).

For all four patients, exon‐spanning copy number variants (CNVs) were predicted from the exome sequencing results using ExCopyDepth (Samarakoon et al. 2014) and the predicted CNVs were filtered and annotated using cnvScan (Samarakoon et al. 2016).

### Variant evaluation

For the Oslo samples, FILTUS (Vigeland et al. 2016) was used for variant filtering and prioritization. Rare, high‐quality variants were selected based on NHLBI Exome Sequencing Project (ESP), 1000 Genomes (1000 Genomes Project Consortium et al., 2012), Exome Aggregation Consortium (ExAC), and in‐house generated databases. The in‐house database in Oslo contains 950 exomes from patients with various disorders (non‐PIDD) such as global developmental delay, intellectual disability, and neurodegenerative diseases. The BCM‐HGSC in‐house database contains exomes from >10,000 individuals (ARIC, Atherosclerosis Risk in Communities) (Gambin et al. 2015) and from > 5000 individuals with various genetic diseases and their healthy relatives. The Integrative Genomic Viewer (IGV) (Thorvaldsdottir et al. 2013) was used for visualization and Alamut (v2.7.1) (Interactive Biosoftware, Rouen, France) in the evaluation of potential pathological relevance.

For patient #1, both parents were also exome sequenced, and the results analyzed as a trio, with the expectation of de novo or compound heterozygous inheritance. Patient #2 was analyzed as a singleton. Patients #3 and 4 were analyzed in parallel, assuming the same disease‐causing variants in both.

### Sanger sequencing

Verification and family segregation were performed using Sanger sequencing. Sanger sequencing was performed on genomic DNA extracted from whole blood. Primers were designed using Primer3 software (Rozen and Skaletsky 2000). Sequencing was performed on an ABI 3730 sequencer (Applied Biosystems, Life Technologies, Foster City, CA) and sequence data were analyzed using SeqScape v2.7 (Life Technologies, CA).

### Ortholog alignment

*CARMIL2* orthologs were downloaded using the UniprotKB module of the UniProt database (Uniprot Consortium, 2015). Manual curation of the downloaded protein sequences, keeping only the longest transcript for all species, resulted in orthologs from 36 different species, including human. The orthologs were aligned using Jalview (Waterhouse et al. 2009)(v.2.8.2), running MuscleWC with default parameters.

### Autozygosity mapping and haplotyping

For patients #1 and 2, autozygosity mapping was performed directly from the vcf files, using the AutEx algorithm in FILTUS (Vigeland et al. 2016). AutEx uses a hidden Markov model to approximate the identity by descent (IBD) as inferred by the absence of heterozygosity (AOH) along each chromosome, and incorporates the genetic distance between variants, allele frequencies, and genotype uncertainty. Before running AutEx, low‐quality variants were removed by applying suitable filters (PASS; DP>9; GQ>39, genomicSuperDups=NA).

**Figure 2:**
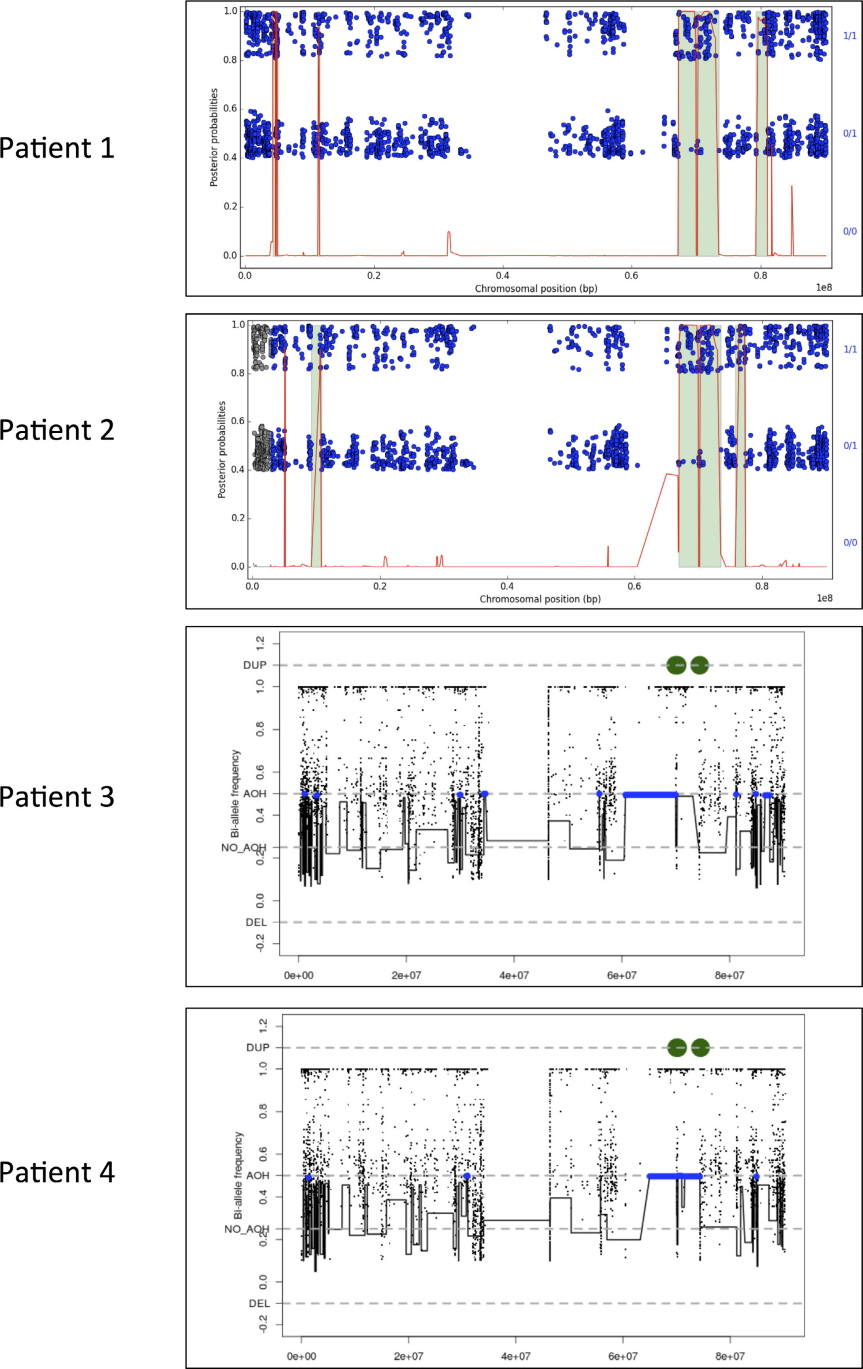
Autozygosity mapping of chromosome 16, based on the exome sequencing results for patients #1 and 2, and SNP array for patients #3 and 4. For patients #1 and 2, the plots were made in FILTUS, and show the posterior autozygosity probability for each variant against its chromosomal position (red curve). Regions called as autozygous are marked in green. Single variants are shown as blue dots: homozygous variants in the upper band; heterozygous in the lower. For patients #3 and 4, autozygosity mapping was performed using exome data. Regions called as autozygous are marked in blue.

For patients #3 and 4, autozygosity mapping was done using the results from the cSNP array (Illumina Infinium Human Exome v1‐2 array) performed as part of the exome sequencing quality control assessment. This assessment included orthogonal confirmation of sample identity and purity using the error rate in sequencing (ERIS) pipeline developed at the BCM‐HGSC. Using an “e‐GenoTyping” approach, ERIS screens all sequence reads for exact matches to probe sequences defined by the variant and position of interest. A successfully sequenced sample must meet quality control metrics of ERIS SNP array concordance (>90%) and ERIS average contamination rate (<5%). The cSNP array allowed a low‐resolution genome‐wide scan to detect large CNVs and regions of AOH. To identify regions of AOH, first we transformed the original B‐allele frequency data by subtracting 0.5 and taking the absolute value for each data point. Next, we applied the circular binary segmentation algorithm implemented in DNAcopy (Olshen et al. 2004) and segments with a mean signal >0.45 and size > 1 Kb were classified as AOH regions. Suspected AOH regions from the cSNP array data were independently evaluated using the exome data and a similar procedure (B‐allele frequency was estimated by calculating the ratio of variant reads/total reads for each SNV from vcf file) in the same region (Fig. 2). The region of homozygosity was confirmed by Sanger sequencing 14 SNVs in the delimiting region in the four patients (Table S1, primer sequence provided on request).

### Flowcytometry

B‐ and T‐cell subpopulations were analyzed by flow cytometry. For B‐cell analysis, the blood samples were washed twice before incubation with antibodies. T‐cell analysis was performed on unwashed blood samples. Briefly, EDTA‐blood was incubated with optimally titrated antibodies for 15 min at room temperature, followed by erythrocyte lysis using BD FACS Lysing Solution (Beckman Dickinson, San Jose, CA). Data acquisition was performed on a Gallios Flowcytometer (Beckman Coulter, San Diego, CA). For T cells, 1 × 10^5^ cells were acquired and for B cells, 1 × 10^6^ if possible. The following antibodies were used: CD31, CD45RO, CD28, CD45RA, CD127, CD19, CD27; Becton Dickinson, CD4, CD8, CD3, CD25, CD38, IgM, IgD; Beckman Coulter, TCR alfa/beta, CD21; R&D Systems (Minneapolis, MN), CXCR5; eBioscience (San Diego, CA), CD45; Invitrogen (Waltham, MA), CD27; Dako (Glostrup, Denmark). The following subpopulations were determined: B cells were gated as CD19+ and further subclassified as naive (IgD+, IgM+, CD27‐), IgM memory (CD27+, IgD+, IgM+), class switched (CD27+, IgM‐, IgD‐), plasmablasts (CD19+dim, CD27++, CD38++), transitional (IgM++, CD38++, CD24+), and CD21 low B cells (CD38 low, CD21 low). T cells were gated as CD3+ and further as naive CD4+ (CD4+, CD45RA+), recent thymic emigrants (CD4+, CD45RA+, CD31+), CD4+ memory (CD4+, CD45RO+), follicular like CD4+ (CD4+, CD45RO+, CCR5+), regulatory T cells (CD4+, CD25++, CD127‐), naive CD8+ (CD8+, CD27+, CD28+), CD8+ early effector memory (CD8+, CD27+, CD28‐), CD8+ late effector memory (CD8+, CD27‐, CD28‐).

For the measurement of intracellular cytokines, whole blood (Na‐Heparin) was diluted 1:2 (RPMI) and stimulated with PMA for 4 h in the presence of ionomycin, brefeldin A, and monensin (Cell stimulation Cocktail plus protein transport inhibitor; eBioscience). Surface staining of CD45, CD3, CD4, and CD8 was done before fixation and permeabilization (fix‐perm) and staining of intracellular IFN*γ* (BD) in T cells.

For intracellular IL‐17 analysis in T cells, whole blood was stimulated for 20 hrs with PMA, brefeldin, and monensin (Protein Transport Inhibitor Cocktail; eBioscience) was added and the samples stimulated for 4 h. Staining was done as above for surface antigens, and staining of intracellular IL‐17 was done after fixation and permeabilization using anti‐IL17A (eBioscience). Since CD4 is downregulated upon stimulation to varying degrees, CD4+ T cells were gated indirectly as CD45+, CD3+, CD8‐ cells. NK cells were gated as CD45+, CD3‐, CD56+. Isotype controls were used to correct for autofluorescence. A control sample from a healthy blood donor was included in all assays.

FoxP3 measurement was done according to the manufacturer's instructions (Human Regulatory T‐cells whole‐blood staining kit; eBioscience) with a few modifications to optimize the method. Briefly surface staining for CD3, CD25, CD4, and CD45 was done before lysing of erythrocyte, fixation, and permeabilization (4**°**C, 30 min). Mab FoxP3 (clone PCH101) was added and incubated at 4**°**C for 60 min. A normal control was run in parallel. Acquisition was done on a Canto II flowcytometer (Beckman Dickinson).

Data analysis for all the above assays was done using Kaluza (Beckman Coulter) and Infinicyte (Cytognos, Salamanca, Spain) software.

### Homology modeling

The three‐dimensional structure of the leucine‐rich repeat (LRR) of CARMIL2 was modeled using the Phyre2 protein fold recognition server (Kelley et al. 2015). On the basis of the crystal structure of mouse CARMIL residues 1‐668 (PDB accession number 4K17) (Zwolak et al. 2013), which was identified as the top rank template, residues 154–702 of human CARMIL2 comprising the LRR domain was modeled with 100% confidence.

## Results

With a common phenotype of warts and other skin symptoms, *CXCR4*,* DOCK8*,* GATA2*,* MAGT1*,* MCM4*,* RHOH, STK4*,* TMC6,* and *TMC8* were candidate genes for all subjects. Exome sequencing did not identify any potential disease‐causing SNPs, indels, or CNVs in any of these genes. However, a homozygous variant in *CARMIL2* (NM_001013838, c.1916T>A, p.Leu639His) was identified in all four subjects. Sanger sequencing confirmed homozygosity in all four affected, heterozygosity in four parents (of patients #1, 3, and 4), and heterozygosity or wild‐type in three unaffected siblings (of patients #1, 3, and 4) (Fig. 1). DNA from the parents of patient #2 was not available.

The affected amino acid p.Leu639His is not present in ESP or dbSNP (Sherry et al. 2001)(May 2016). It is, however, reported in ExAC in the heterozygous state in seven of 24,979 patients of European (non‐Finnish) origin (MAF 0.0001401). The in‐house database in Oslo contains two heterozygote carriers of the variant and none in the BCM‐HGSC in‐house databases BHCMG and ARIC.

### Autozygosity mapping and haplotyping

The three families are of Norwegian origin, but without known consanguinity within or between families. Thus, a homozygous disease‐causing variant was unexpected in all families. This, and the presence of the variant in heterozygote state in the European ExAC cohort raised the question of the variant being a founder mutation in the Norwegian population. Therefore, haplotype analysis was performed to investigate whether the *CARMIL2* variant is located on an identical haplotype on both alleles.

In all four patients, haplotyping indicated that the *CARMIL2* variant c.1916T>A is located within a region with AOH (Fig. 2). The boundaries of the AOH were fine‐mapped by collecting all SNVs in the region from the exome sequencing results (Table S1). All variants were manually curated by visual inspection in IGV (Thorvaldsdottir et al. 2013). Furthermore, to avoid considering reference genome errors when defining the AOH boundaries, only homozygous variants with an allele frequency <0.8 in ExAC and/or 1000 Genomes were included in the analysis. All heterozygous variants passing manual curation were included. Sanger sequencing of 14 SNVs was performed to identify the AOH regions (Table S1). Definition of the AOH boundaries was complicated by the presence of sequence with high homology to other genomic regions, as sequencing reads mapping to several genomic locations can produce false‐positive variants. Sanger verification of these variants was also hampered by difficulties in designing specific primers.

Using the exome results (Table S1), the AOH region covering *CARMIL2* was calculated to span between 5.7 and 7.3 Mb for patient #1 (Table 2). The two affected siblings, patients #3 and 4, were observed to have identical AOH regions, spanning 12.8–14.1 Mb. For patient #2, the AOH starts at the same loci as patients #3 and 4, and ends at the same loci as patient #1, spanning 11.3–14.1 Mb. The common AOH for all four affected patients equals the region reported for patient #1. Using Decode's map of the human genome (Kong et al. 2010), FILTUS estimates the common region to span between 1.05 and 1.09 centiMorgans, thus indicating a distant familial relationship. No other shared AOH regions were detected.

**Table 2:** AOH regions surrounding the *CARMIL2* variant in the three affected families. The regions are shown by genomic coordinate and size of the AOH (Mb)

Patient	Genomic coordinates (hg19) for AOH region (chr16)	AOH region size (Mb)
1	(67180171_67292201)_(72993860_74485788)	5.7–7.3
2	(60393236_61686848)_(72993860_74485788)	11.3–14.1
3 and 4	(60393236_61686848)_(74501856_74504005)	12.8–14.1

Positioning of the *CARMIL2* variant within an AOH common for all four patients, together with the observation of the variant in the European cohort of ExAC, indicates that this variant may be a founder mutation in the Norwegian population.

### Immunophenotyping

Flow cytometry of blood cells from the affected patients revealed strikingly similar immunological characteristics (Table 3). All four patients had markedly reduced levels of FoxP3‐expressing regulatory T cells, most prominent in the two sisters (patients #3 and #4). While the absolute numbers of CD4+ cells was well within the normal range for all patients, there was a clear increase in the proportion of CD4+ naive cells, and a reduction in CD4+ memory and follicular CD4+ cells. Furthermore, CD4+ cells in all patients were characterized by low expression of IFN*γ*, in particular, in patients # 2, 3, and 4. Interestingly, a similar pattern was seen within the NK cell compartment, with nearly normal absolute counts but reduced IFN*γ* expression. Three of the four patients had low levels of Th17 cells, most clearly demonstrated in patient #2. The total number of B cells was in the normal range for all patients, but characterized by features of B‐cell pathology with low levels of class‐switched B cells and plasmablasts in all but one patient.

**Table 3:** Immunological features of the four patients with homozygous *CARMIL2* mutation

Flow cytometry	Reference values	Patient 1	Patient 2	Patient 3	Patient 4
CD3 (cells/*μ*L)	800–2400	1649	3972	2020	1743
CD8 (cells/*μ*L)	200–1000	495	464	310	248
CD4 (cells/*μ*L)	500–1400	1046	3497	1659	1402
CD16 (cells/*μ*L)	100–400	474	89	140	168
CD19 (cells/*μ*L)	100–500	572	440	411	348
CD4+memory (%)	41–84	35	23	22	28
CD4+follicular‐like (%)	6,2–18	2.4	1.1	1.9	1.2
CD4+ naive (%)	25–71	83	94	92	93
RTE (%)	28–72	47	56	50	57
CD8+naïve (%)	34–87	72	32	82	62
CD8+ early effector/ memory (%)	2.9–16	22	10	10	11
CD8+ late effector/ memory (%)	2.6–58	6	57	6	26
TReg (CD25++, CD127−) (%)	2.5–5.8	0.5	2.4	0.5	0.8
TReg (FoxP3) (%)	2.1–7.4	0.7	1.3	0.3	0.2
Naive B cells (%)	48–83	95	83	97	90
IgM‐memory B cells (%)	3.3–22	2.5	6.1	1.6	5.3
Class‐switched B cells	4.3–23	1.9	5.5	1	3.8
Transitional B cells (%)	0.6–4.6	4	1	7.7	14.4
Plasmablasts (%)	0.3–5.1	0.1	0.4	0.1	0.1
CD21 low B cells (%)	1.2–9.4	0.9	1.7	0.6	0.2
Th17 (%)	>0.6%	1.43	0.05	0.4	0.54
CD4+ IFN*γ* + (%)	>5%	5.2	1.35	1.8	3.3
CD8+ IFN*γ* + (%)	>14%	14.7	35	10	18
CD56+ IFN*γ* + (%)	>49%	62	33	21	22

Deviations from reference values are colored by: red (reduced levels), orange (level borderline deviation), and blue (increased levels).

RTE, Recent thymic emigrants.

## Discussion

The four patients presented in this paper all have a history where immunodeficiency and an immunodysregulated phenotype was strongly suspected, although no definitive diagnosis was given prior to exome sequencing. The identification of a homozygous missense variant in *CARMIL2* led to further investigation and scrutiny of their history, highlighting several common clinical and phenotypic traits (Table 1).

All four patients had common skin findings that in addition to warts included molluscum contagiosum and various forms of dermatitis from early childhood. Gastrointestinal disease, childhood asthma, herpes group viral infections, respiratory tract infections, and cystitis, in one caused by BK virus infection, were observed to a variable degree. Clinically, there is a clear susceptibility to viral infections and three of the four patients (#2, 3, and 4) have low levels of both IFN*γ*‐producing NK cells and CD4+ T cells, known to predispose to recurrent and persistent viral infection. Forthcoming studies should characterize their NK cell function. Patient #2 is distinguished by frequent cutaneous fungal infections and notably he has markedly reduced numbers of Th17 cells known to be important in the defense against skin and mucosal fungal infection. Patient #4 has hypogammaglobulinemia corresponding with a clear propensity for bacterial respiratory tract infections. This is not evident in the others, although patients #1 and 3 have a low percentage of class‐switched CD19+ B cells associated with hypogammaglobulinemia.

The suspicion of an immunodeficiency was raised by the observed increased risk of viral infections in these patients, and further strengthened by the observed immunodysregulation affecting skin and mucosa. They display variable degree of aphthous stomatitis and other mucosal ulcers without identification of any viral agents, as well as inflammatory bowel disease. Furthermore, their psoriatic‐like skin lesions and dermatitis point to a hyperinflammatory response well known in other forms of immunodeficiency.

The leiomyosarcoma found in patient #3 is a rare malignancy that has been associated with EBV infections and HIV infection. However, although both she and her affected sister have had recurrent infections of two other members of the herpes virus group, notably HSV and varicella zoster virus (VZV), she has not shown any signs of recurrent or chronic EBV infections. Immunophenotypically, all affected subjects had decreased Treg counts, known to be associated with autoimmunity and inflammation including gastrointestinal manifestations. Again, further studies will try to characterize Treg function in these patients. Notably, although all affected individuals have the same disease‐causing variant, variable expression is observed, even between the two sisters (patients #3 and 4).

The exact function of *CARMIL2 (*also known as *RLTPR)* is not known. *CARMIL2* is one of three genes in the human CARMIL family, which have high sequence homology and are important to cell migration, but are known to have distinct cellular functions (Liang et al. 2009; Edwards et al. 2014). *CARMIL2* encodes a 1435 amino acid protein, which includes four known motifs: an RGD domain, a leucine‐rich repeat (LRR), a tropomodulin domain, and a proline‐rich region (Matsuzaka et al. 2004). The gene was first identified and characterized as downregulated in affected psoriatic tissue (Matsuzaka et al. 2004), pointing to a potential role in regulation of skin pathology. Investigations of protein expression have shown general cytoplasmic expression with additional expression in immune cells (Uhlen et al. 2015).

**Figure 3:**
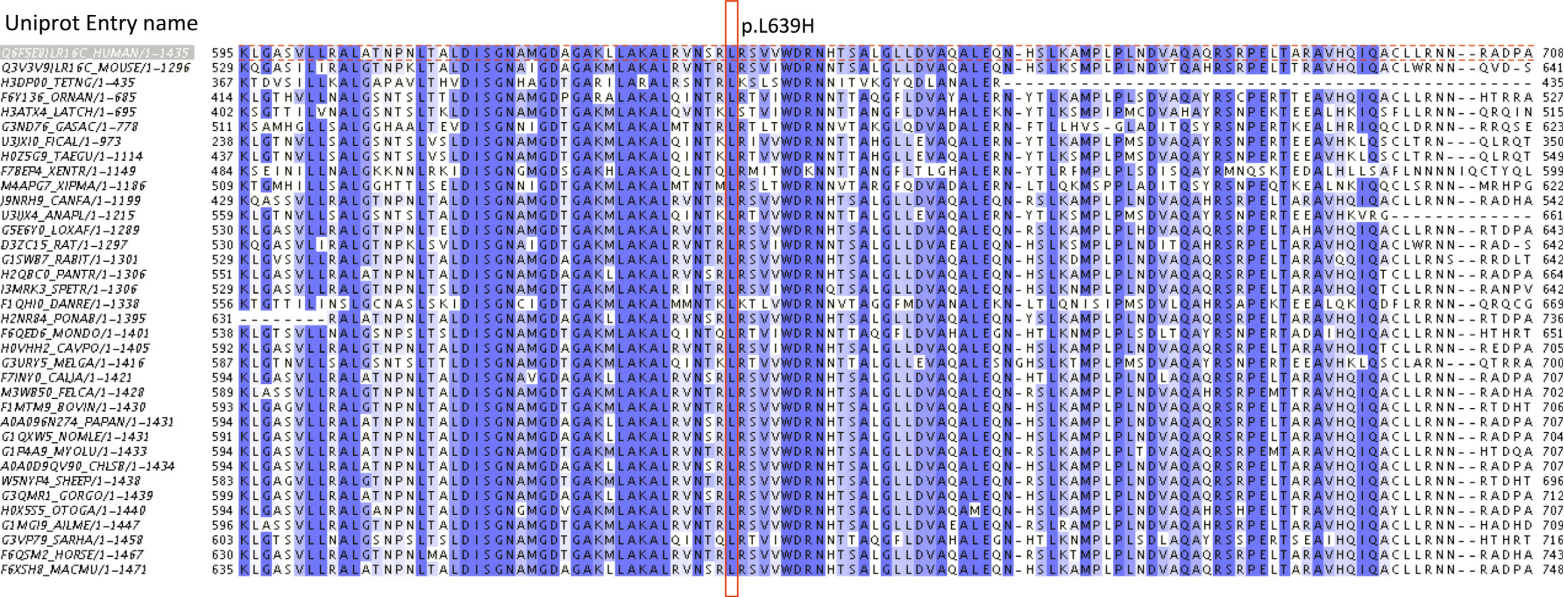
Alignment of *CARMIL2* orthologs from 36 species using Jalview (v.2.8.2) running Muscle WC with default parameters. Each entry is annotated by UniProt entry name and number of amino acids. The alignment was colored by percentage identity. Dark blue bars indicate amino acids with high level of conservation between species, white bars indicate lower level of conservation.

The affected amino acid p.Leu639 is located within the LRR domain, and is highly conserved between species (Fig. 3). The LRR domain is a widespread structural motif that is commonly involved in protein–protein interactions. LRR domains have a consensus sequence motif of LxxLxLxxN/CxL (Kobe and Deisenhofer 1994; Kajava et al. 1995) and the main contribution to the stability of LRR domains are thought to come from the hydrophobic inner core containing these conserved leucines (Bella et al. 2008).

**Figure 4:**
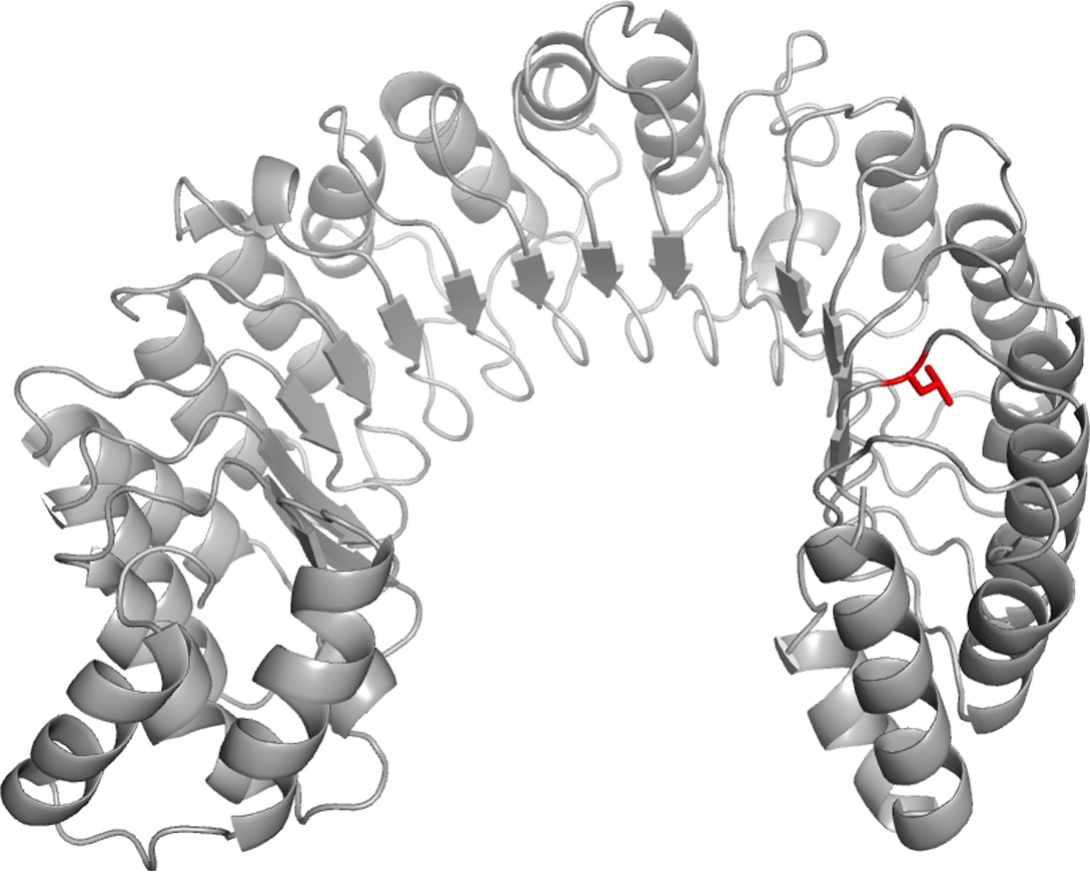
Homology model of the LRR domain in *CARMIL2*, showing the location of p.Leu639 (depicted in red) in LRR 15. LRR, leucine‐rich repeat

A homology model of the LRR domain in *CARMIL2* obtained using the Phyre2 Protein Fold Recognition Server (Kelley et al. 2015) shows that the modeled LRR domain has an overall planar horseshoe shape (Fig. 4). The domain is capped at the N‐ and C‐terminal ends by helix‐loop‐helix motifs that are highly conserved among CARMIL isoforms (Zwolak et al. 2013). The affected amino acid p.Leu639 (depicted in red) lies in LRR 15 (of total 16) and corresponds to the first leucine in the canonical LRR sequence (LxxLxLxxN/CxL) where it contributes to the hydrophobic core region between the helices and the sheets. Thus, changing the hydrophobic amino acid leucine to the more polar and titratable histidine may potentially destabilize the three‐dimensional structure and hence disrupt the function of the LRR domain. The crucial role of the first leucine in the canonical LRR sequence has also been indicated in a study of disease‐causing mutations reported in different genes with LRR domains, where variants affecting this position have frequently been reported to cause defects in protein function and disease (Matsushima et al. 2005).

Functional testing has been reported of a Rltpr variant (p.L432P) in mice (Liang et al. 2013). This variant affects the highly conserved leucine in position 6 of the canonical LRR sequence of LRR 8 (of total 16 LRRs in mice), and functional testing indicated that this alteration may lead to a disruption of Rltpr's ability to link CD28 to PKCθ and Carma1. This study suggests that *Rltpr* has an essential role in the development of regulatory T cells by costimulation via CD28 by controlling its internalization (Liang et al. 2013). Further, knock‐out studies of *Rltpr* in mice have shown a phenotype of decreased regulatory T cells and reduced proliferative and IL‐2‐secretion response to anti‐CD3 and anti‐CD28 antibodies (Eppig et al. 2015). This study also suggests a role for *CARMIL2* affecting Treg, Th17 cells, IFN*γ*‐producing CD4+ T cells as well as NK cells, known to be of importance for autoimmunity, fungal infections in the skin and mucosa, and defense against viral infection, respectively.

In conclusion, the four reported patients were all homozygous for the same highly conserved, missense variant in *CARMIL2* and the specific variant may represent a Norwegian founder variant segregating on a population‐specific haplotype. In addition, they shared several common clinical traits and immunological findings, which were consistent with perturbation of the reported biological functions of CARMIL2. Overall, the presented data support defective function of CARMIL2 as causing the disease observed in these three families.

## Conflict of Interest

J.R.L. has stock ownership in 23andMe, is a paid consultant for Regeneron Pharmaceuticals, has stock options in Lasergen, Inc., is a member of the Scientific Advisory Board of Baylor Genetics, and is a co‐inventor on multiple United States and European patents related to molecular diagnostics for inherited neuropathies, eye diseases, and bacterial genomic fingerprinting. The Department of Molecular and Human Genetics at Baylor College of Medicine derives revenue from the chromosomal microarray analysis (CMA) and clinical exome sequencing offered at Baylor Genetics (BMGL:http://www.bmgl.com/BMGL/Default.aspx).

## Supporting information


**Table S1.** Variants deviating from the reference sequence (hg19) in the region surrounding the *CARMIL2* variant on chromosome 16. Position, reference allele, frequency in ESP and 1000 Genomes Project Consortium et al. (2012), alternative allele and genotype, as detected by exome sequencing, is shown for the four affected patients. The autozygous regions are marked in green. Some heterozygous variants are observed within the autozygosity region, and these are marked in orange. The heterozygous SNVs in *PDXDC2P* and *PDPR* are likely due to *PDXDC2P* being a pseudogene with a high sequence homology to *PDXDC1*, and *PDPR* having a pseudogene: *LOC283922. LOC283922* is located in the region defining the downstream boundary of the AOH, and thus, heterozygous SNVs are observed in the downstream region as well. Furthermore, the succeeding gene in the downstream region, *NPIPB15* belongs to the nuclear pore complex interacting protein family, which have a high internal sequence homology. The alignment problems caused by these pseudogenes are also indicated by the low mapping quality of the reads covering these regions. Variants confirmed by Sanger sequencing are marked in blue. The genotypes of the Sanger‐confirmed variants are also shown for parents of patients #1, 3, and 4, and for three healthy siblings in the same two families.
